# Making MRI available for patients with cardiac implantable electronic devices: growing need and barriers to change

**DOI:** 10.1007/s00330-019-06449-5

**Published:** 2019-11-27

**Authors:** A. N. Bhuva, R. Moralee, J. C. Moon, C. H. Manisty

**Affiliations:** 1 Department of Cardiac Imaging, Barts Heart Centre, Barts Health NHS Trust, West Smithfield, London, EC1A 7BE UK; 2 Institute for Cardiovascular Science, University College London, London, UK

**Keywords:** Pacemaker, artificial, Defibrillators, implantable, Magnetic resonance imaging

## Abstract

**Abstract:**

More than half of us will need a magnetic resonance imaging (MRI) scan in our lifetimes. MRI is an unmatched diagnostic test for an expanding range of indications including neurological and musculoskeletal disorders, cancer diagnosis, and treatment planning. Unfortunately, patients with cardiac pacemakers or defibrillators have historically been prevented from having MRI because of safety concerns. This results in delayed diagnoses, more invasive investigations, and increased cost. Major developments have addressed this—newer devices are designed to be safe in MRI machines under specific conditions, and older legacy devices can be scanned provided strict protocols are followed. This service however remains difficult to deliver sustainably worldwide: MRI provision remains grossly inadequate because patients are less likely to be referred, and face difficulties accessing services even when referred. Barriers still exist but are no longer technical. These include logistical hurdles (poor cardiology and radiology interaction at physician and technician levels), financial incentives (re-imbursement is either absent or fails to acknowledge the complexity), and education (physicians self-censor MRI requests). This article therefore highlights the recent changes in the clinical, logistical, and regulatory landscape. The aim of the article is to enable and encourage healthcare providers and local champions to build MRI services urgently for cardiac device patients, so that they may benefit from the same access to MRI as everyone else.

**Key Points:**

• There is now considerable evidence that MRI can be provided safely to patients with cardiac implantable electronic devices (CIEDs). However, the volume of MRI scans delivered to patients with CIEDs is fifty times lower than that of the estimated need, and patients are approximately fifty times less likely to be referred.

• Because scans for this patient group are frequently for cancer diagnosis and treatment planning, MRI services need to develop rapidly, but the barriers are no longer technical.

• New services face logistical, educational, and financial hurdles which can be addressed effectively to establish a sustainable service at scale.

## Introduction

**Fig. 1:**
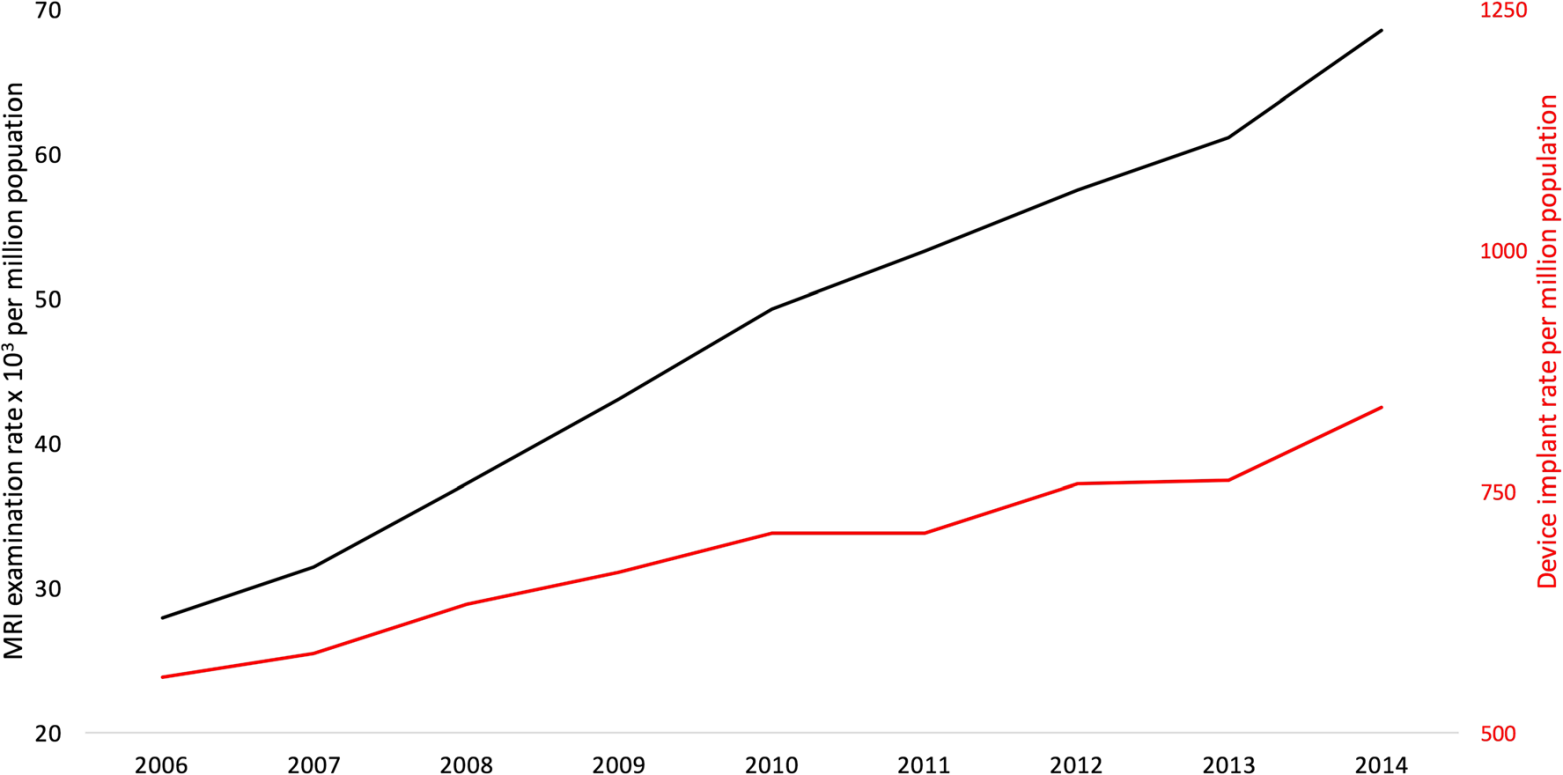
Annual total device implant rate (red) and MRI scan provision (black) in England [2, 3]

Magnetic resonance imaging (MRI) is fundamental to healthcare, particularly for cancer diagnosis and treatment (surgery, radiotherapy), for diseases of the central nervous and musculoskeletal systems. Sixty million scans are performed annually worldwide [1], and it is the fastest growing imaging modality at 12% annually (Fig. 1) [2]. MRI is beginning to replace invasive biopsy for diagnosis of some cancers and is now often essential for planning of neurosurgical procedures and CyberKnife radiotherapy [4].

However, one in 50 over the age of 75 has a permanent pacemaker (PPM) or implantable cardioverter–defibrillator (ICD)—these are collectively termed cardiac implantable electronic devices (CIEDs) [3, 5]. Historically, MRI has been contraindicated for safety but each of these individuals has an estimated need for MRI in their lifetime of 50–75%, particularly because they are older and have more comorbidities [6]. Now, technical developments have made it reasonable to perform MRI for these individuals [7], but provision remains grossly inadequate due to logistical difficulties. This review therefore discusses the significant non-technical barriers to developing a sustainable service.

## Safety

Overall, MRI is an extremely safe imaging modality, with over 300 million scans performed worldwide to date [8, 9]. There have been reported safety events in patients who did not undergo CIED reprogramming prior to an MRI scan [10]. Most likely, this was because radiology teams were not aware of the presence of a CIED, because it was not detected by staff or volunteered by the patient. To the authors’ knowledge, there have been no deaths attributed to the performance of MRI in patients with CIEDs when performed intentionally, with the correct protocols followed. In patients with CIEDs, the risk has been attributed to the interaction between magnetic field and device components [11]. Adequate patient monitoring and safety procedures are therefore paramount, and so scanning guidelines for CIED patients have recently been published in the UK, Europe, and the USA [12–16].

## New technology

To address the safety issues, an industry-wide effort was needed to develop MRI-conditional devices [17]. Software modifications with an “MRI mode” were introduced so re-programming before and after scanning became straightforward and electromagnetic interference minimized. Since their approval in 2008, multiple studies and over 10,000 scans have demonstrated their safety, and are detailed elsewhere [7, 12].

If an MRI-conditional generator is combined with MRI-conditional leads from a different manufacturer, however, this renders the system non MRI-conditional because these components have not been tested together. Despite little clinical evidence of risk associated with non MRI-conditional leads undergoing MRI, increased perception of risk leads to difficulties with access for these patients [18].

## Non MRI-conditional CIEDs

The majority of patients with CIEDs currently in situ however still have non MRI-conditional “legacy” devices implanted. The situation is rapidly changing for scanning these devices and professional guidelines are now endorsing scanning if there is a clear indication after an individual risk–benefit assessment [12, 14]. The evidence base is large with two studies showing that the risk of scanning legacy pacemakers and defibrillators is tiny. Provided strict protocols under close medical supervision were adhered to, there have been no major events in 3600 scans where programming was performed correctly [15, 16]. The risk is particularly low when compared with a major complication rate of 0.4 to 2% associated with elective laser-assisted lead extraction to replace a legacy system with an MRI-conditional CIED [19, 20]. In a prospective registry (the REPLACE registry), the rate of major complications among patients undergoing generator replacement with or without the placement of an additional transvenous lead was 4 to 15% [21]. It is however difficult to prove that MRI scanning in all circumstances is completely safe—there are an almost infinite number of lead–generator combinations that would require testing. For the patient and healthcare system, the test should be whether it is safer to scan than not to scan. Fortunately, major societies now provide protocols related to MRI scanning in patients with non MRI-conditional devices [12, 14, 22].

## Where are we at the moment?

Patients with CIEDs and clinicians however have reported severe difficulties accessing MRI scans (even for those with MRI-conditional devices), with requests often declined without clear reason [23]. The scale of the problem is large, but hard to quantify—requests are censored by patients and referrers, as well as by the lack of service provision. US data suggests that patients with CIEDs are between 40 and 50 times less likely to be referred for MRI than they should be, and refusal rates for non-clinical reasons (logistical/financial/access) remain high [24, 25]. Similar experience has been reported in Ireland, Italy, and the UK, with 73% reporting delays in receiving appointments [26–29]. In an Italian prospective questionnaire study, 39% of patients with CIEDs were denied MRI scans. Fifty percent of whom reported being denied because they had a CIED implanted, even though MRI-conditional [28].

## An ignored and growing health inequality

We start from a low level of provision, and major barriers still exist. A national audit covering 86% of hospitals in England reported there were less than 1000 scans performed annually and less than half of units will scan MRI-conditional devices [26]. An estimated 50,000 MRI scans are needed a year in the UK for patients with CIEDs, meaning this is a 50-fold under-provision (Supplementary Data) [2, 3]. MRI capacity required is similar to other countries with reported data. Estimates in the USA are for 200,000 scans a year in a patient population four times the size, consistent with prospective European data [28, 30]. Bridging this gap would expand total MRI activity by around 2% (current annual growth for MRI services is at 12%) [2]; however, it is likely to be offset by reduced activity in other areas.

**Fig. 2:**
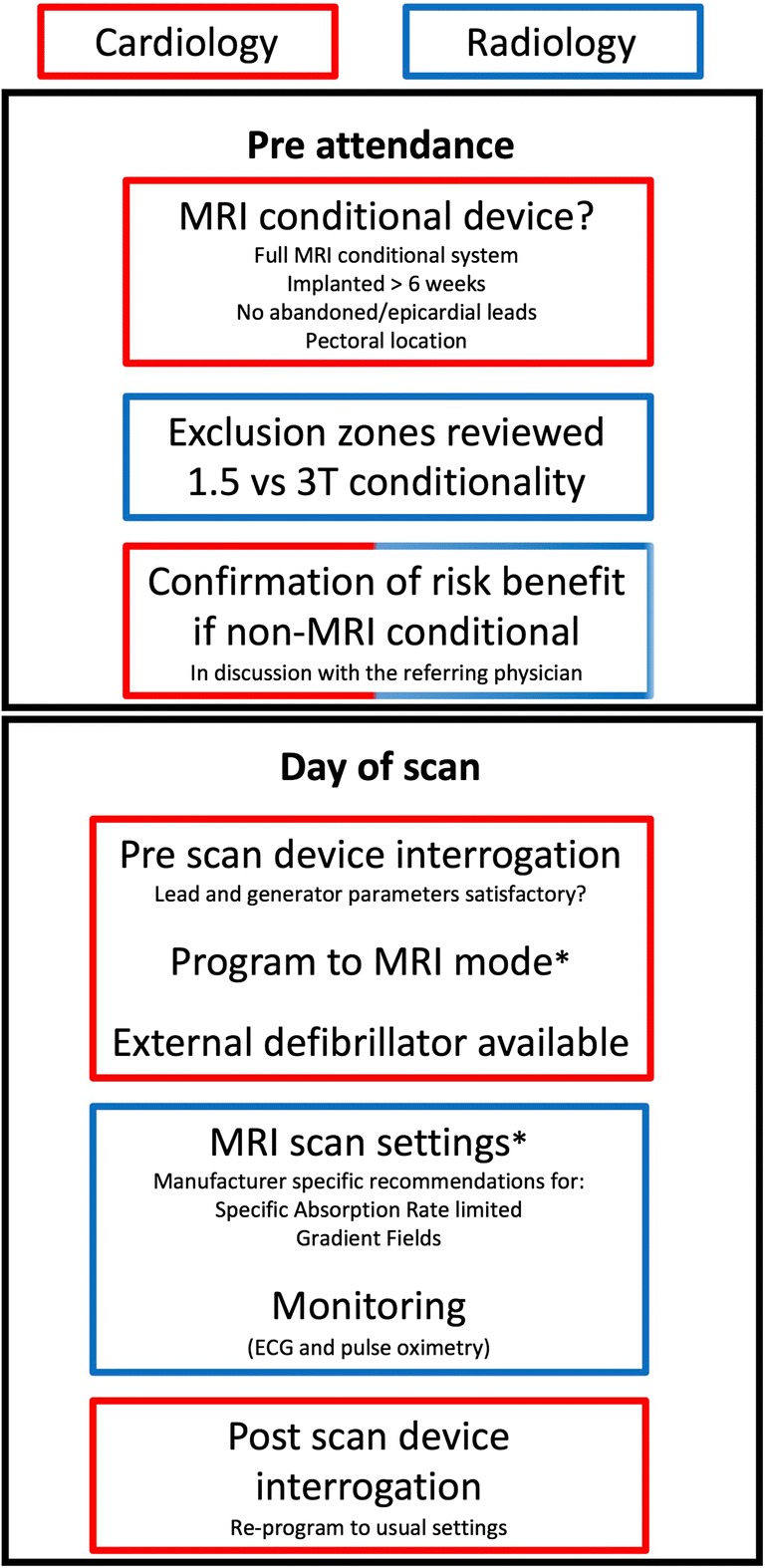
Logistical steps to provide MRI for patients with CIEDs. Manufacturer-specific guidances are available for MRI-conditional devices available at mrimypacemaker.com. At steps indicated by an asterisk symbol, follow published guidelines for Non MRI-conditional devices [12, 16]

The need is growing by 10–15% a year (Fig. 1). Alongside increasing demand for MRI, CIED implantation is rising by 5% per year. Half of this group is aged over 65 years with increased comorbidities and therefore will rely on optimal diagnostic imaging [5]. Because equipment to perform these scans is available in most MRI departments [26], it should mean that these scans are now provided and the need is met. The focus of the remainder of the review is to understand the remaining barriers; however, it is first necessary to understand the extra logistical safety steps needed that give rise to these (Fig. 2).

## Protocols for MRI in patients with CIEDs

Before the scan, it is necessary to check whether the CIED is indeed MRI-conditional—with both leads and generator comprising part of an MRI-conditional system. This can be done via referrers, patient records, device identification cards (given to the patient at the time of implantation), or the radio-opaque markers on leads and pulse generators seen on chest x-ray. This often presents the greatest obstacle to scanning, as patients have their devices implanted in different clinical facilities to where their MRI is requested. An increasing number of patients now hold details of their device themselves, which can facilitate this process. Some older, previously non-MRI-conditional leads and generators have now been retrospectively tested and approved as MRI-conditional. This means that labelling of a device as non MRI-conditional cannot be taken as a definitive, permanent categorization, potentially leading to confusion for clinicians and patients alike. Each device manufacturer offers an MRI device check tool in order to assess the suitability of an individual patient’s device components to address this issue, centralized at mrimypacemaker.com. Non MRI-conditional devices require that a clear indication is established through discussions between clinicians and patient, and protocols under close supervision are followed. Increasingly, there are centers that scan almost all patients with CIEDs, meaning that the practice of checking device conditionality prior to scanning can be streamlined.

Before the scan (usually on the same day), the device is checked and programmed using a portable unit by a cardiologist or cardiac physiologist. For patients with MRI-conditional devices, this involves straightforward re-programming to “MRI-Safe” mode, and for those patients with legacy devices, this involves manual re-programming. There is also a safety checklist to identify patients deemed higher risk for undergoing MRI scanning. The presence of an epicardial or abandoned lead may be an exclusion at a particular institution, but this type of patient is not addressed by current recommendations. There is a growing understanding of the risks and risk mitigation in these scenarios, so patients are not systematically excluded [31]. Overly rigid selection protocols may therefore cause patients more harm (from not scanning) than good.

During the scan, the patient should be monitored using both electrocardiography and pulse oximetry. An advanced cardiac life support–trained person should be present for the duration of the scan, regardless of MRI-conditionality [12]. An external defibrillator with pacing capability should be immediately available. On MRI completion, the device must be interrogated and restored to the initial settings—either in the department or scanner side.

## MRI scanning conditions

CIEDs are only MRI-conditional under specific controls of the MRI environment intended to lower power. This includes the region of imaging, field strength, spatial gradients, and specific absorption rate (SAR). Specific conditions can be found in manufacturer device check tools. The use of SAR may be unreliable in the presence of a CIED, and so manufacturers are moving towards reporting a more precise measure of energy deposition, the root mean square value of the B1 field (B1+RMS) [32].

## MRI artifact

Diagnostic imaging can be limited due to metallic artifact, but this is usually restricted to cardio-thoracic scans. Artifact is related to the type and proximity of the device, being more prominent in ICDs than PPMs due to the greater off-resonance induced by the battery and high-voltage transformer. Strategies to reduce artifact generally yield interpretable results and so ensure clinical yield is high, which is particularly important for patients with ICDs [33]. These strategies include using spoiled gradient echo cines, shorter echo times, or wideband inversion recovery pulses for late gadolinium enhancement [34, 35].

## An early diagnosis makes clinical and economic sense

When MRI scans are provided for CIED patients, about a third are for acute indications and another third yield a positive cancer diagnosis [36]. The impact on decision-making is remarkably high [33, 37], but is limited to the experience of a few centers [26]. The consequences of not performing these scans can be devastating, particularly for cancer, half of transient ischemic attacks or spinal cord compression—where delayed diagnosis and travelling great distances for an MRI scan are particularly unwarranted [29]. Change must therefore be implemented rapidly.

The benefit is evident both for the patient and healthcare system. Timely scanning is important for all these indications and has a financial benefit in addition to the patient care—a prompt cancer diagnosis for example saves $5000, and an extra inpatient bed-day costs $300 [38, 39]. Medical bodies and health economists recognize this need [40], and successive reports have highlighted the clinical and financial costs of a late diagnosis [41].

## Barriers to scale and what we need

Referrers and service providers still perceive safety concerns. One survey assessing current practice attributed this perception to other hurdles including logistical problems, a lack of inter-disciplinary support, poor education, and re-imbursement [26]. Reluctance to develop services has been observed in many countries [27]. Information to check device compatibility is disparate and requires accessing different manufacturer guidelines and cardiology and radiology protocols. This means centers lack confidence because information is not easily accessible and adds on time to preparation and scanning. Already busy services therefore have valid concerns about managing rising demand. Many patients with legacy devices find it difficult to accept that they can undergo MRI scanning, as they have been told repetitively in the past that they can never undergo this. Re-education is therefore required at every level of an institution—from the patient to the cardiologist, as well as referring physicians/surgeons and radiology departments.

Scanner and device manufacturers are working to break down some of these barriers. The latest pacemakers can be pre-programmed days in advance to anticipate a scan and will sense the MRI environment to switch in and out of MRI-mode when the patient enters and leaves the scan room [42]. This avoids MRI department workflows being disrupted by reliance on cardiac device physiologists being available at the same time as the MRI. Some pacemakers have a portable hand-held activator that can be activated by the patient pre-scan [42]. While this provides some flexibility, it remains necessary to interrogate devices after MRI to ensure correct function. MRI manufacturers are also introducing software packages to simplify adjustments to scanner settings [43]. Such technological aids are appreciated by clinicians but refining care pathways to minimize the multi-disciplinary workflow represents the larger preceding hurdle. Even when strict protocols are followed, complications are rare, making this a resource-intensive but low-yield risk reduction strategy.

Locally, it has been useful to centralize expertise in a few named individuals, including administration staff, within each department. A standardized and specific referral form that is completed by referrers prior to booking a scan helps to reduce delays. MRI scans for patients with CIEDs have also been redesigned into dedicated lists, whereby all necessary staff are present. This however involves reaching across standard silos of practice in a hospital, but facilitates easy decision-making and improving confidence (particularly at the early stages of setting up a service) [29].

## Current progress

It will soon be indefensible not to provide this service, and 2018 represented a year of policy change. The Centers for Medicare and Medicaid Services recently changed funding guidance for non MRI-conditional CIEDs and now states there is sufficient evidence for the coverage of MRI in CIEDs that do not have FDA labelling, unless there is a fractured or abandoned lead [44]. The UK Royal College of Radiology and British Cardiovascular Society released a joint statement recognizing the need to develop more services [45]. The statement acknowledges the devastating consequences of not undergoing MRI and the growing health inequality. This represents high-level consensus to develop new working practices and partnerships and should de-risk hospitals from establishing new services. Legal ambiguity however still remains a problem. As noted by the German Cardiac and Roentgen Societies, apportioning responsibility between radiology and cardiology departments can make establishing inter-disciplinary services complex [46]. In Italy, contradicting laws both implicitly allow and explicitly forbid MRI scanning in this setting at the same time [23].

Remarkably, hospitals frequently implant an MRI-conditional CIED, spending the extra money but do not offer to scan it. Cardiology standards are now addressing this, beginning to explicitly ask CIED implantation centers to ensure pacing support for MRI units [47]. The 2018 British Heart Rhythm Society Standards for Implantation and Follow-up of Cardiac Rhythm Devices state: “Each device centre must ensure that they have agreements and arrangements in place that allow their patient’s access to MRI scanning. Patients should not be denied access to MRI scanning because of lack of these arrangements or resource.” Implementing an MRI service for CIED patients is therefore now an imperative for radiology and cardiology practices. An important next step will be to establish billing arrangements that recognize the scan complexity.

## Conclusion

Clinical demand for MRI in patients with these devices is high; however, provision remains poor. Change must now be rapid to resolve this for patients who need diagnosis and treatment planning of otherwise terminal pathology. Addressing this will require education of clinicians, referrers, and patients alongside strategies for streamlining workflows, improving re-imbursement, and developing new collaborations.

## Abbreviations

CIED: Cardiac implantable electronic devices
ICD: Implantable cardioverter–defibrillator
MRI: Magnetic resonance imaging
PPM: Permanent pacemaker
SAR: Specific absorption rate
